# Tubulinopathies continued: refining the phenotypic spectrum associated with variants in *TUBG1*

**DOI:** 10.1038/s41431-018-0146-y

**Published:** 2018-04-30

**Authors:** Stefanie Brock, Katrien Stouffs, Emmanuel Scalais, Marc D’Hooghe, Kathelijn Keymolen, Renzo Guerrini, William B. Dobyns, Nataliya Di Donato, Anna C. Jansen

**Affiliations:** 1 Faculty of Medicine, Vrije Universiteit Brussel, Brussels, Belgium; 2 Centre for Medical Genetics, UZ Brussel, Brussels, Belgium; 3 Neurogenetics Research Group, Reproduction Genetics and Regenerative Medicine Research Cluster, Vrije Universiteit Brussel, Brussels, Belgium; 4 Division of Pediatric Neurology, Department of Pediatrics, Centre Hospitalier de Luxembourg, Luxembourg City, Luxembourg; 5 Department of Neurology, Algemeen Ziekenhuis Sint-Jan, Bruges, Belgium; 6 Pediatric Neurology Unit and Laboratories, Children’s Hospital A. Meyer, University of Florence, Florence, Italy; 7 IRCCS Stella Maris Foundation, Pisa, Italy; 8 Center for Integrative Brain Research, Seattle Children’s Research Institute, Seattle, WA USA; 9 Department of Pediatrics (Genetics), University of Washington, Seattle, WA USA; 10 Department of Neurology, University of Washington, Seattle, WA USA; 11 Institute for Clinical Genetics, TU Dresden, Dresden, Germany; 12 Pediatric Neurology Unit, Department of Pediatrics, UZ Brussel, Brussels, Belgium

## Abstract

Tubulinopathies are a heterogeneous group of conditions with a wide spectrum of clinical severity resulting from variants in genes of the tubulin superfamily. Variants in *TUBG1* have been described in three patients with posterior predominant pachygyria and microcephaly. We here report eight additional patients with four novel heterozygous variants in *TUBG1* identified by next-generation sequencing (NGS) analysis. All had severe motor and cognitive impairment and all except one developed seizures in early life. The core imaging features included a pachygyric cortex with posterior to anterior gradient, enlarged lateral ventricles most pronounced over the posterior horns, and variable degrees of reduced white matter volume. Basal ganglia, corpus callosum, brainstem, and cerebellum were often normal, in contrast to patients with variants in other tubulin genes where these structures are frequently malformed. The imaging phenotype associated with variants in *TUBG1* is therefore more in line with the phenotype resulting from variants in *LIS1* (a.k.a. *PAFAH1B1*). This difference may, at least in part, be explained by gamma-tubulin’s physiological function in microtubule nucleation, which differs from that of alpha and beta-tubulin.

## Introduction

The gamma-tubulin protein (TUBG1) was first described by Oakley and Oakley [1], and the encoding *TUBG1* gene has later been mapped to chromosome 17q2 [2–4]. *TUBG1* shares 94.6% of nucleotides and 97.3% of amino acids with its paralog *TUBG2*, but despite this high degree of similarity they appear not to be functionally synonymous. Both *TUBG1* and *TUBG2* are highly expressed in the developing and mature human brain, including the cerebral cortex, cerebellum, thalamus, and hippocampus, with *TUBG1* being expressed more abundantly than *TUBG2* [5].

Variants in genes belonging to the tubulin superfamily, including *TUBA1A*, *TUBB2A*, *TUBB2B*, *TUBB3*, *TUBB*, and *TUBG1*, have been associated with a spectrum of cortical malformations through disruption of normal microtubule interactions, which are involved in neuronal cell proliferation, migration and differentiation, as well as axon growth and guidance [6–15]. Microtubule nucleation precedes the formation of bipolar spindles and separation of chromosomes in mitosis, steps that are necessary for the progression of the cell cycle [16, 17]. Unlike alpha-tubulin and beta-tubulin, gamma-tubulin is not incorporated in the microtubule lattice but is required for the polymerization of the alpha-tubulin and beta-tubulin proteins. Therefore, gamma-tubulin localizes to the centrosome during interphase. This process is mediated by protein kinases [18]. Two gamma-tubulin proteins associate with gamma complex proteins 2 and 3 (GCP2, GCP3) to form a gamma-tubulin small complex (γTuSC). Binding of GCP4, GCP5, and GCP6 to several copies of γTuSC results in the formation of a γ-tubulin ring complex (γTuRC). γTuRC links microtubules to the spindle pole during mitosis [19]. Insufficient proliferation due to defective microtubule function can ultimately lead to microcephaly [8]. Defects in neuronal proliferation and migration are linked to the role microtubules play in cell shape and orientation [20].

Protein structures within the tubulin superfamily show a high degree of similarity. However, the phenotypic differences associated with variants in the various tubulin isotypes support the hypothesis that each tubulin has a distinctive function [21].

An alanine-scanning mutagenesis screen of human gamma-tubulin in *S. pombe* showed that all deleterious variants in the *TUBG1* gene were found in residues predicted to be located at the surface, some in positions to interact with alpha and/or beta-tubulin at the microtubule lattice. The localization of these variants might therefore indicate domains within the protein that are responsible for gamma-tubulin’s individual function [22, 23].

To date, three unrelated patients with de novo variants in *TUBG1* have been reported [14]. Two had microcephaly and bilateral symmetric pachygyria with a posterior to anterior gradient on imaging. They suffered from spastic quadriparesis and were bedridden. The third patient presented with a milder phenotype with normal head circumference, mild intellectual disability, and posterior pachygyria. The corpus callosum was malformed in all three patients, whereas the basal ganglia, the cerebellum, and the brainstem were spared. All three patients presented with seizures in early life.

We report the identification of eight additional patients from seven families with four novel heterozygous variants in *TUBG1*, contributing to a further delineation of the associated clinical and imaging phenotype.

## Methods

### Patient samples

Patients were recruited through the international research network of the authors. The study was approved by the Institutional Review Board of the UZ Brussel (B.U.N. 143201214360), the Institutional Review Board at Seattle Children’s Hospital, and the Pediatric Ethics Committee of the Tuscany Region. Informed consent was obtained from all families prior to genetic studies. Clinical data were collected through clinical examination and review of medical records. All imaging data were reviewed by AJ, NDD, and WBD.

Blood samples for DNA preparation and genetic investigation were obtained with informed consent from patients and parents. DNA was extracted using standard protocols.

For patients 1 and 2, variant analysis was performed using gene panel analysis. This analysis was performed at the Center of Medical Genetics, UZ Brussel in collaboration with the Brussels Interuniversity Genomics High Throughput core (BRIGHT core) according to standard procedures (see http://www.brightcore.be/). Raw data are quality controlled by use of FastQC (v0.10.1) and mapped to the human reference genome with BWA 0.7.10. Mapping qualities are assessed via overall coverage analysis by an in-house designed script. The mapped reads are processed using the GATK 2.7.2 (Genome Analysis Toolkit) pipeline (IndelRealaginer, BaseRecalibrator, HaplotypeCaller) and the detected variants are annotated by Annovar or Alamut Batch.

Patients 3, 4, and 7 were studied using targeted panel sequencing with Single-Molecule Molecular Inversion Probes as previously described (PMID: 27773430) [24]. For patients 5 and 6 whole-exome sequencing (WES) was done at the Broad Institute Genomic Services using Agilent SureSelect enrichment kit with subsequent sequencing of the libraries on a HiSeq 2000 (Illumina, San Diego, CA, USA). Sequence reads were aligned to the human genome (hg19) using BWA software or the CLC Biomedical Genomics workbench. Downstream processing was done with the Genome Analysis Toolkit, SAMtools, and Picard Tools. Single-nucleotide variants and indels were subsequently called by GATK Unified Genotyper (PMID:21478889) [25] and a variant quality score of ≥10 and were annotated using SeattleSeq SNP annotation and Annovar. Variants were then filtered using standard hard-filtering parameters (PMID:21478889) [25]. Specifically, only variants with a quality score of ≥30, sequencing depth of ≥10, quality/depth ratio of ≥5, length of homopolymer run of ≤5.0, and allelic balance of ≤0.80 were considered for downstream analysis.

For patient 8, WES was performed at the French National Centre for Genotyping (Evry, France). Library preparation, exome enrichment, WES, and analysis of variants were performed as previously described [26]. Exome sequencing quality data were homogeneous with an average mean depth higher than 100×. Coverage depth greater than 15× and 5× was obtained for about 97 and 99% of the target, respectively. We analyzed variants affecting coding regions and essential splice sites and excluded all variants with frequencies higher than 1% in multiple genome databases including the Single-Nucleotide Polymorphism Database, 1000 Genomes, the National Heart, Lung, and Blood Institute Exome Variant Server, the Exome Aggregation Consortium (ExAC), and a local Paris Descartes Bioinformatics platform database. The c.776C>T p.(Ser259Leu) variant was confirmed by Sanger sequencing and shown to be de novo. The reference sequence used was NM_001070.4 with systematic numbering of the exons (1–11); or as described in NG_033886.1. All variants have been submitted to https://databases.lovd.nl/shared/genes/TUBG1 (patient ID 00134040-00134047).

## Results

We report four novel heterozygous variants in *TUBG1* in eight patients, including two siblings. Clinical and imaging findings are summarized in Table 1.

**Table 1:** Clinical and imaging features associated with variants in *TUBG1*

Clinical features											
	Patient 1	Patient 2	Patient 3	Patient 4	Patient 5	Patient 6	Patient 7	Patient 8	Patient 9 (LIS-TUB-027)	Patient 10 (LIS-TUB-028)	Patient 11 (LIS-TUB-029)
Reference	This report	This report	This report	This report	This report	This report	This report	This report	Poirier et al. [14]	Poirier et al. [14]	Poirier et al. [14]
Sex	M	M	F	F	F	M	M	F	F	F	M
Nucleotide sequence variation	c.63C>A	c.985G>T	c.776C>T	c.776C>T	c.776C>T	c.776C>T	c.769A>T	c.776C>T	c.991A>C	c.1160T>C	c.275A>G
Protein sequence variation	p.(Phe21Leu)	p.(Asp329Tyr)	p.(Ser259Leu)	p.(Ser259Leu)	p.(Ser259Leu)	p.(Ser259Leu)	p.(Ile257Phe)	p.(Ser259Leu)	p.(Thr331Pro)	p.(Leu387Pro)	p.(Tyr92Cys)
Mode of inheritance	de novo	Father's DNA n/a	de novo	de novo	Germline mosaicism in parent	de novo	de novo	de novo	de novo	de novo	de novo
Age at examination	33y	21y	19mo	14y	11y 6mo	9y 6mo	15y	18mo	31y	21y	18mo
Head circumference (SD)	57 cm	53.1 cm (<−2.6SD)	<−3.5SD	n/a	47.5 cm at 6y 6mo (<−3.3SD)	n/a	51.3 cm at 13y (<−2.5SD)	n/a	Normal	<−5.5SD	<−4SD
Dysmorphic features	No	No	No pictures	No pictures	No pictures	No pictures	No	No	n/a	n/a	n/a
Congenital anomalies	No	No	No	No	Strabismus	No	No	No	Cataract	n/a	n/a
Intellectual disability	Severe	Severe	n/a	n/a	Moderate	Moderate	Moderate (FS IQ-score 44)	Severe	Moderate ID	Severe ID	Severe ID
Motor impairment	Spastic quadriplegia; walks with support	Spastic quadriplegia	Delayed motor development	Unsteady gait	Spastic diplegia	n/a	n/a	Delayed motor development	Moderate CP	Spastic quadriplegia	Spastic quadriplegia
Speech and language development	No speech, only sounds	Non-verbal	Delayed	Non-verbal	Speaks 50 words	Non-verbal	Speakes 5–6 word sentences	Non-verbal	n/a	n/a	n/a
Other	Assisted feeding	Gastrostomy	–	–	Drooling	–	–	–	–	–	–
Epilepsy	Yes	Yes	No	Yes	Yes	Yes	Yes	Yes	Yes	Yes	Yes
Age at seizure onset	36m	n/a	–	6m	4m	n/a	3y 11mo	5m	n/a	n/a	n/a
Seizure type	Tonic–atonic–myoclonic	Partial complex: versive seizure, myoclonic	–	Tonic-clonic	Generalized tonic-clonic	n/a	n/a	Focal, versive	Early onset epilepsy	Early onset epilepsy	Infantile spasms
Refractory	n/a	Yes	–	n/a	n/a	n/a	n/a	No	Yes	Yes	Yes
Imaging features											
Age at MRI	36y	11y	1y 6mo	12mo	13y 7mo	2mo	6y	9y	n/a	n/a	n/a
Gyral pattern	Pachygyria over the posterior frontal lobe and parieto-occipital cortex	Agyria, diffuse	Pachygyria diffuse, mild over frontal lobes, moderate posterior, cortex 10–13 mm	Pachygyria diffuse, cell sparse zone over occipital lobes, cortex 13–15 mm	Pachygyria diffuse, mild over frontal lobe, and moderate over temporal and occipital lobes, cortex 6–13 mm	Pachygyria, diffuse, mild over frontal lobe, and moderate over temporal and occipital lobes, cortex >15 mm	Pachygyria, nearly normal cortex over frontal lobes, pachygyria over perisylvian and occipital lobes, cortex 6–10 mm	Pachygyria diffuse, mild over frontal lobe, moderate over temporal > occipital lobes, bilateral deep infolding parietal	Pachygyria, nearly normal cortex over frontal lobes, moderate over perisylvian and occipital lobes	Pachygyria diffuse, mild over frontal lobe, and moderate over temporal and occipital lobes	Pachygyria diffuse, mild over frontal lobe, and moderate over temporal and occipital lobes
Gradient	P>A	P>A	P>A	P>A	P>A	P>A	P>A	P>A	P>A	P>A	P>A
White matter	Enlarged perivascular spaces	Severely reduced	Mildly reduced	Mildly reduced	Normal	Normal	Mildly reduced	Mildly reduced	Normal	Mildly reduced	Severely reduced
Lateral ventricles	Enlarged posterior horns	Severly enlarged	Mildly enlarged	Mildly enlarged	Enlarged posterior horns	Mildly enlarged	Mildly enlarged posterior horns	Mildly enlarged	Normal	Mildly enlarged	Mildly enlarged
Corpus callosum	Normal	Thin	Normal	Normal	Normal	Thin	Normal	Thin	Dysmorphic, thick	Thin	Dysmorphic, thick
Basal ganglia	Normal	Dysplastic	Normal	Normal	Normal	Normal	Normal	Dysplastic	Normal	Normal	Normal
Hippocampus	Malrotation	n/a	Normal	Normal	Normal	Normal	Normal	Normal	n/a	n/a	n/a
Brainstem	Normal	Hypoplasia	Normal	Normal	Normal	Normal	Normal	Normal	Normal	Normal	Normal
Cerebellum											
Cortex	Normal	Normal	Normal	Normal	Normal	Normal	Normal	Normal	Normal	Normal	Normal
White matter	Normal	Normal	Normal	Normal	Normal	Normal	Normal	Normal	Normal	Normal	Normal
Vermis	Normal	Hypoplasia	Normal	Normal	Normal	Normal	Normal	Normal	Normal	Normal	Normal

RefSeq NM_001070.4A anterior, *CP* cerebral palsy, *F* female, *FS IQ* full-scale IQ, *ID* intellectual disability, *M* male, *mo* months, *n/a* not available, *P* posterior, *SD* standard deviation, *y* years, – absent

At last examination patients were aged 18 months to 33 years. They all had moderate to severe intellectual disability, including very limited or absent language development, and variable degrees of motor impairment ranging from delayed motor development to severe spastic quadriplegia. Head circumference was available for five patients, one of whom was normocephalic and four were microcephalic (<−2.5SD). None of the patients showed dysmorphic facial features; one had congenital strabismus. Except for patient 3, all patients presented with seizures with a variable time of onset between the first months of life till after the 3rd year of life.

**Fig. 1:**
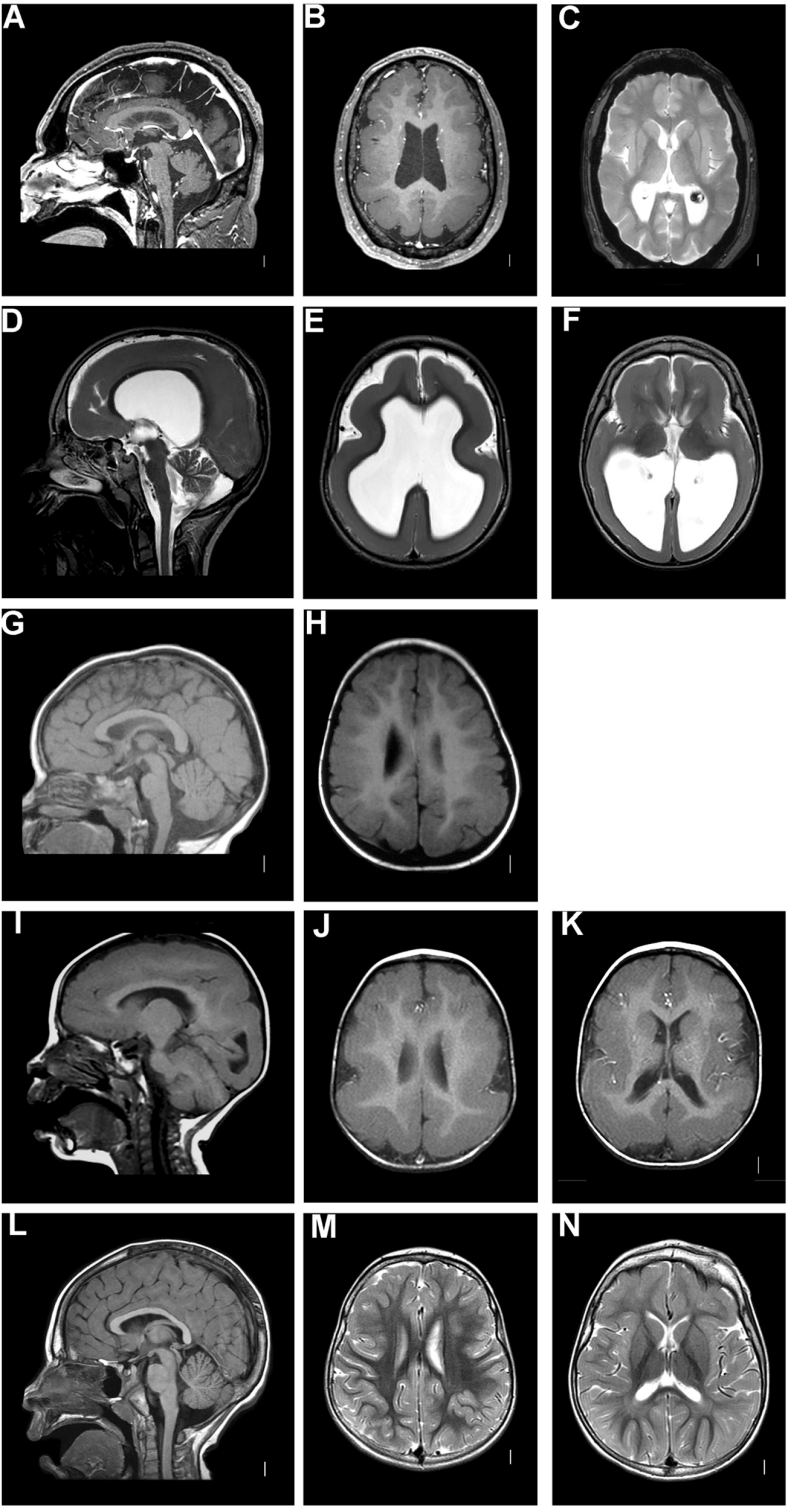
Imaging characteristics patients 1–4 and a normal subject. Brain MRI of patient 1 at age 36 years. Sagittal planes through the midline (**a**) show a normal corpus callosum, brainstem, and cerebellum. Axial T1-weighed images (**b**) and T2-weighed images (**c**) show pachygyria with a posterior to anterior gradient, enlarged posterior horns of the lateral ventricles, and enlarged perivascular spaces. Brain MRI of patient 2 at age 11 years. Sagittal planes (**d**) show a thin corpus callosum, hypoplasia of the brainstem, and the cerebellar vermis. Axial T2-weighed images (**e**, **f**) show diffuse lissencephaly, reduced white matter, and enlarged lateral ventricles and dysplastic basal ganglia. Brain MRI of patient 3 at age 1 year 6 months (**g**, **h**) and patient 4 at age 12 months (**i**, **j**, **k**). Sagittal planes (**g**, **i**) show a normal appearance of corpus callosum, brainstem, and the cerebellum. Axial T1-weighed images (**h**, **j**, **k**) show pachygyria with a posterior to anterior gradient, enlarged lateral ventricles, and reduced white matter. Brain MRI of a healthy subject (age 4 years). Sagittal image (**l**). Axial T2-weighed images (**m**, **n**)

**Fig. 2:**
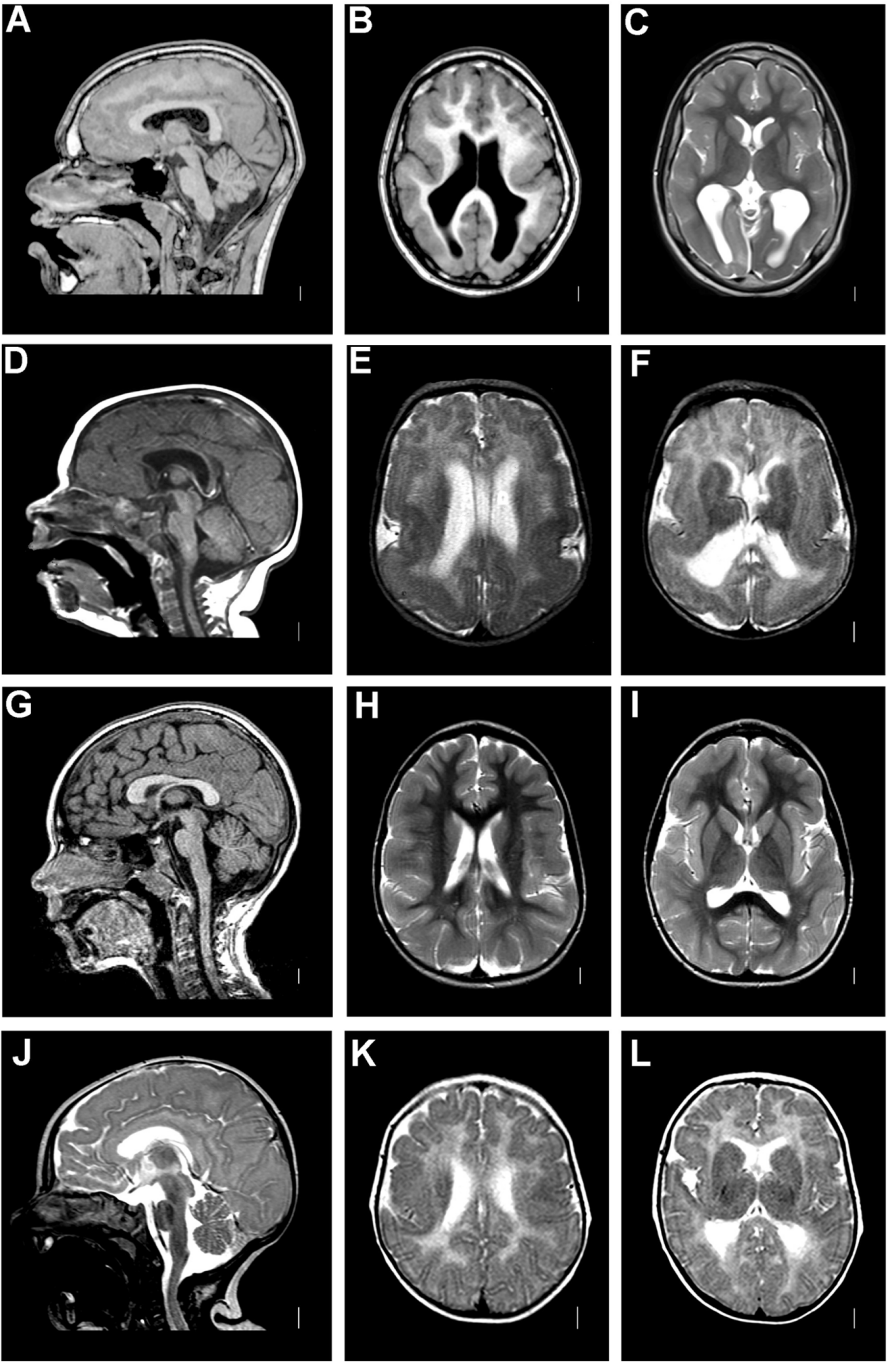
Imaging characteristics patients 5–8. Brain MRI of patient 5 at age 13 years. Sagittal planes through the midline (**a**) show the absence of malformations of the corpus callosum, brainstem, and cerebellum. Axial T1-weighed images (**b**) and T2-weighed images (**c**) show pachygyria with a posterior to anterior gradient and enlarged posterior horns of the lateral ventricles. Brain MRI of patient 6 at age 2 months. Sagittal planes (**d**) show a thin corpus callosum, absence of hypoplasia of the brainstem, or the cerebellar vermis. Axial T2-weighed images (**e**, **f**) show diffuse pachygyria with a posterior to anterior gradient and enlarged lateral ventricles. Brain MRI of patient 7 at age 1 year 6 years. Sagittal images (**g**) show a normal appearance of corpus callosum, brainstem, and the cerebellum. Axial T2-weighed images (**h**, **i**) show pachygyria with a posterior to anterior gradient with almost normal frontal lobes, enlarged posterior horns of the lateral ventricles, and reduced white matter. Brain MRI of patient 8 at age 9 years. Sagittal images (**j**) show hypoplasia of the corpus callosum. Axial T2-weighed images (**k**, **l**) show pachygyria with a posterior to anterior gradient and important involvement of the temporal lobes, bilateral parietal infoldings, dysplastic basal ganglia, enlarged lateral ventricles, and reduced white matter

Brain magnetic resonance imaging (MRI) studies were performed between ages 2 month and 36 years (Figs. 1, 2). The cerebral cortex was most abnormal in patient 2 who had diffuse agyria. The other seven patients, five of which harbored the same c.776C>T variant, had a relatively homogeneous phenotype on imaging studies, characterized by diffuse pachygyria with a posterior to anterior gradient. The white matter showed a variable reduction in volume in six patients. Basal ganglia were dysplastic in patients 2 and 8. Brainstem and cerebellum were normal in all except for patient 2, who had brainstem and cerebellar vermis hypoplasia.

**Fig. 3:**
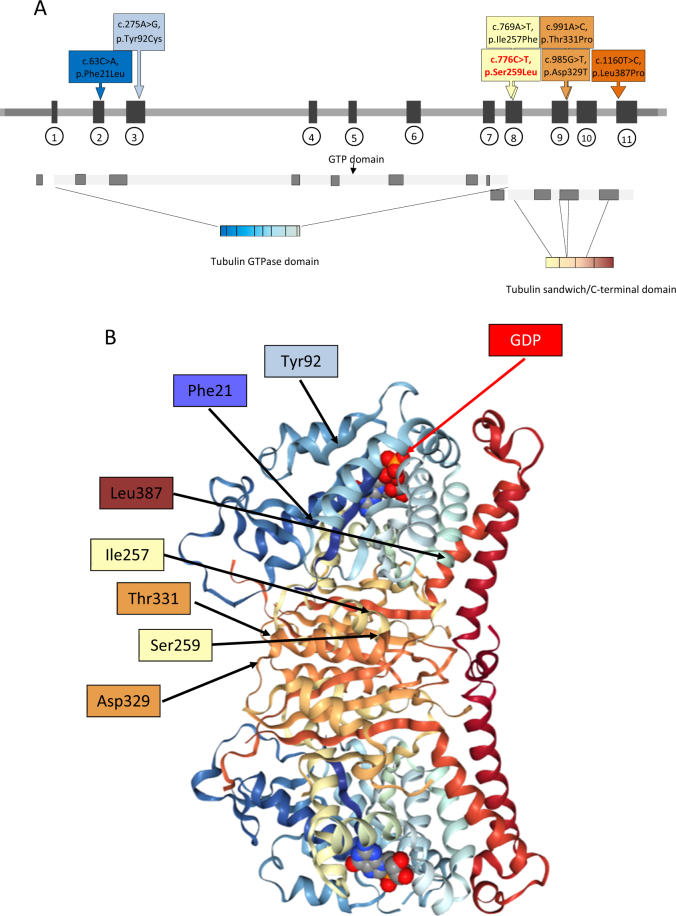
Distribution of the variants in the *TUBG1* gene. Linear (**a**) and 3D (**b**) representation of the *TUBG1* gene showing its functional domains and the distribution of the described *TUBG1* variants. The variant in red in **a** represents the recurrent variant detected in four patients. The 3D structure is based on PDB ID: 3CB2 (crystal structure of gamma-tubulin bound to GDP) using NGL viewer [38, 39]

NGS analysis identified four novel heterozygous missense variants in *TUBG1* (Table 2). Patients 3, 4, 5, 6, and 8 all share the same c.776C>T, p.(Ser259Leu) variant. In the siblings (patients 5 and 6), the variant was inherited by one of the parents who carries a germline mosaicism of this variant. Patient 7 is heterozygous for the c.769A>T, p.(Ile257Phe) variant, which is located very close to the recurrent c.776C>T variant. Currently, the c.776C>T variant has not been described in any database. Prediction programs SIFT, MutationTaster, PolyPhen2 all predict that this highly conserved nucleotide/amino acid is deleterious (Table 2). This variant is located in the Tubulin/FtsZ 2-layer sandwich (or C-terminal) domain (Fig. 3).

**Table 2:** Overview of identified variants (RefSeq: NM_001070.4) and PolyPhen, SIFT, and MutationTaster scores

Patient ID	1	2	3–6	7	8
DNA	c.63C>A	c.985G>T	c.776C>T	c.769A>T	c.776C>T
Protein	p.(Phe21Leu)	p.(Asp329Tyr)	p.(Ser259Leu)	p.(Ile257Phe)	p.(Ser259Leu)
Exon	2	9	8	8	8
MAF	–	–	–	–	–
ExAC	–	–	–	–	–
PolyPhen2 (0 = neutral ⇨ 1 = deleterious)	0.935	1.000	0.928	1.000	0.928
SIFT (1 = neutral ⇨ 0 = deleterious)	1	0	0.04	0	0.04
Mutation-Taster (0 = neutral ⇨ 1 = deleterious)	1	1	1	1	1
Align GVGD (C0 = neutral ⇨ C65 = deleterious)	C0	C35	C15	C15	C15

*ExAC* exome aggregation consortium, *MAF* minor allele frequency, *PolyPhen2* polymorphism phenotyping v2, *SIFT* sorting intolerant from tolerant, “–” indicates that no data are available

## Discussion

Tubulinopathies have been characterized by a broad range of cortical malformations associated with hypoplasia or agenesis of the corpus callosum, dysmorphic basal ganglia, and hypoplasia of the brainstem, cerebellar vermis, and/or hemispheres [6, 9, 11, 27]. In some patients, the cerebellar dysgenesis and/or dysplastic basal ganglia may be more prominent than the cortical malformations [28].

Poirier et al. [14] initially described the phenotype associated with variants in *TUBG1* as similar to that associated with variants in *LIS1* (a.k.a. *PAFAH1B1*); an observation that has recently been integrated in the classification of lissencephalies proposed by DiDonato et al. [11]. A later report on the same patients mentioned that the two individuals with microcephaly and severe pachygyria resembled individuals with lissencephaly carrying the p.(Arg402Cys) substitution in *TUBA1A* [6].

In the current series including eight additional patients with variants in *TUBG1*, the most common imaging phenotype consists of partial or diffuse pachygyria with a posterior to anterior gradient, similar to the phenotype associated with variants in *LIS1*, *DYNC1H1*, or *KIF5*. However, the most severe end of the *TUBG1*-related spectrum also includes diffuse agyria as illustrated by patient 2, similar to the phenotype associated with some variants in *LIS1* or the c.1205G>A, p.(Arg402His) variant in *TUBA1A*. The cortical malformation in patients with variants in *TUBG1* therefore clearly stands out from that of tubulin-related dysgyria, which can be seen in patients with variants in *TUBB2B*, *TUBB3*, *TUBB*, and most variants in *TUBA1A* [11, 29].

In this study, associated brain abnormalities appear to be less frequent compared to other tubulinopathies. Except for two patients, all patients described so far had normal basal ganglia, which is usually considered a key feature for tubulinopathies and has been observed in 75% of patients [28]. Additionally, the brainstem and cerebellum were spared in most patients with *TUBG1* variants, and if malformations in either of these two structures were present, they were usually subtle. This is in contrast to the high prevalence of 78.7% of cerebellar hypoplasia in individuals with variants in other tubulin genes [6, 28]. Polymicrogyria or polymicrogyria-like cortical dysplasia and a simplified gyral pattern, which is common in *TUBB2B* and *TUBB3* variants, have not been described in patients with *TUBG1* variants so far. The observation that *TUBG1* causes undermigration leading to pachygyria or agyria can possibly be explained by a negative impact on cell morphology in patients carrying a *TUBG1* variant, while variants in other tubulin genes are more often associated with dysgyria or overmigration presenting as polymicrogyria which suggests defective radial glial guidance of immature neurons [8].

Thus, the observation that the imaging phenotype associated with variants in *TUBG1* differs from that associated with variants in other tubulin genes is in line with the function of *TUBG1* in nervous system development and its stronger involvement in neuronal migration than in, for example, axon growth and orientation, which appears to be more prominently affected by variants in *TUBB3* [21, 30, 31]. This hypothesis is also supported by the disturbed neuronal migration observed in an in utero RNA interference assay by Poirier et al. [14]. Most patients with variants in *TUBG1* also exhibit microcephaly, indicating a major role of *TUBG1* in neuronal cell proliferation.

It has been shown that tubulin isotypes have individual functions, expression levels, and distribution among different cell types, which led to the assumption that subtle phenotypic differences could exist. As the exact mechanism and the extent of how a variant alters the formation of functional tubulin heterodimers, GTP binding, longitudinal and lateral protofilament interactions, and microtubule interactions with microtubule-associated proteins remains widely unknown, so far, few conclusions can be drawn about these distinctive features [15, 21, 32, 33]. Nevertheless, the differences in physiological function of the *TUBG1* protein as a scaffold in the formation of microtubules on the one hand and alpha-tubulin and beta-tubulin dimers as components of the microtubule on the other hand have been well established, and can give a possible explanation for the different phenotypic presentation on imaging [15].

The correlation between phenotype and genotype could further be determined by the exact location of the variant within the gene. This has been observed in the case of the recurrent c.1205G>A, p.(Arg402His) variant in *TUBA1A* causing classic lissencephaly, and the c.790C>T, p.(Arg264Cys) variant in the same gene associated with central pachygyria [6, 11, 34, 35]. Recurrent *TUBB2B* and *TUBB3* variants have also been described to result in homogeneous phenotypes [15, 30, 36, 37]. This has also been observed in our study as the five patients with the c.776C>T, p.(Ser259Leu) variant shared a similar phenotype including mild frontal and moderate posterior pachygyria with no or subtle malformations of the corpus callosum, brainstem, and cerebellum. Additionally, patient 7 in our study carried a c.769A>T, p.(Ile257Phe) substitution which is only two amino acids upstream of the c.776C>T, p.(Ser259Leu) recurrent variant, and is associated with a similar phenotype. These variants are located at the borderline of the tubulin sandwich/C-terminal domain.

Interestingly, the c.985G>T, p.(Asp329Tyr) variant in patient 2, which was associated with the most severe phenotype in our series is located two amino acids upstream of the c.991A>C, p.(Thr331Pro) variant identified in a patient with posterior pachygyria and a moderate phenotype described by Poirier et al. [14]. These variants are also located in the tubulin sandwich/C-terminal domain. At this moment, it remains hard to predict the phenotype based on the position of the detected variants. However, c.985G>T, p.(Asp329Tyr) is located at the surface of the *TUBG1* protein. In line with findings of the alanine-scanning mutagenesis, variants in surface proteins might have a more severe effect [22]. The majority of the variants are detected in the 2-layered sandwich domain of the *TUBG1* protein, which is probably involved in the formation of dimers (Fig. 3). Consequently, these variants are expected to interfere with the microtubule formation and have a dominant-negative effect on the function of *TUBG1*.

So far, no truncating variants have been described in patients with *TUBG1* variants. Whether truncations are likely to have either more severe phenotypes or no associated phenotype remains unclear. However, the number of variants described remains relatively small to draw conclusions.

Based on the current classification of lissencephalies, the majority of the patients with *TUBG1* variants fit within subtype 2–3. The predicted clinical outcome based on the classification is concordant with the phenotype in our patients [11].

It is not yet possible to identify a particular causative tubulin gene or variant based on clinical and radiologic presentations alone, as findings are not specific enough. However, variants in *TUBG1* should be considered as a possible differential diagnosis in patients presenting with posterior predominant pachygyria with no or minimal involvement of other brain structures, especially if variants in *LIS1* have been ruled out.
